# Elucidating and exploring specialised plant metabolism through a pangenomic and pantranscriptomic lens

**DOI:** 10.1111/tpj.71173

**Published:** 2026-10-21

**Authors:** Nancy Choudhary, Shakunthala Natarajan, Clara Morweiser, Nele Grünig, Marie Hagedorn, Jakob Franke, Boas Pucker

**Affiliations:** ^1^ Institute for Cellular and Molecular Botany (IZMB) University of Bonn Kirschallee 1 53115 Bonn Germany; ^2^ Institute of Botany Leibniz University Hannover Herrenhäuser Str. 2 30419 Hannover Germany; ^3^ Institute of Process Engineering in Life Sciences Karlsruhe Institute of Technology Fritz‐Haber‐Weg 2 76131 Karlsruhe Germany

**Keywords:** biosynthesis, gene clusters, metabolic diversity, presence–absence variation, secondary metabolites, structural variation

## Abstract

Specialised metabolism is an important factor in how plants cope with biotic and, to a lesser extent, abiotic stress. Current approaches to identify and study the genes involved in such metabolic pathways mainly leverage genomic and transcriptomic datasets of plants, in order to improve our understanding of plant resilience. Even though modern sequencing techniques have provided access to high‐quality reference genome or transcriptome datasets, sequencing costs have for a long time forced a restriction to a selected reference accession. However, individual plants within a population often exhibit substantial differences in metabolic profiles, sometimes even leading to completely different chemotypes. This generated an urgent need to transition from single‐reference accessions to the wider diversity of plant populations or even beyond the species level. Thanks to decreasing costs and improved reliability of sequencing techniques, this is now possible through pangenomic or pantranscriptomic approaches. In this review, we provide an introduction into the current state of the fields of pangenomics and pantranscriptomics and highlight recent examples on how these approaches have been used to elucidate and investigate specialised metabolic pathways in plants. We also provide an overview of database resources that might be useful for researchers in the field. Lastly, we present a critical summary and discussion of limitations and trends that are important for future innovations. With these developments in mind, we anticipate that pangenomics and pantranscriptomics will play a crucial role in exploring and harnessing the diversity of plant specialised metabolism beyond single individuals.

## Significance Statement

Past studies of plant specialised metabolism have largely missed the biochemical diversity within plant populations due to being limited to a single‐reference genome or transcriptome. In this review, we summarise current efforts to leverage pangenomic and pantranscriptomic approaches that help to elucidate and capture the full diversity of biosynthetic networks in plants.

## INTRODUCTION

Plants produce a plethora of structurally diverse specialised metabolites that offer evolutionary benefits, particularly for defence against herbivores, pathogens and other forms of stress. Studying the underlying biochemical pathways is highly relevant not only to understand how plants thrive under adverse environmental conditions, but also to develop biotechnological solutions that leverage the metabolic diversity of plants. Modern approaches to elucidate and investigate the pathways of specialised metabolism in plants typically start with the analysis of genome and transcriptome sequences (Jaczkowski et al., 2026). Long‐read genomics has rapidly improved access to the genetic basis of plants (Marks et al., 2021; Schwacke et al., 2025), including the complex biosynthetic networks of specialised metabolism (Hakim et al., 2025; Priego‐Cubero et al., 2025; Reynolds et al., 2026). Sequencing a plant genome has become an almost routine process that can be completed by individual research groups (de Oliveira et al., 2026), which has removed the restriction on model organisms. The two main technologies available for sequencing plant genomes are currently offered by Pacific Biosciences (PacBio) and Oxford Nanopore Technologies (ONT) (Pucker et al., 2022). Nonetheless, in light of the enormous diversity of specialised metabolic pathways, which often exhibit substantial differences between closely related species and even lead to different chemotypes of a single species, there is still a major need to implement further innovations to accelerate the discovery of new biosynthetic pathways and to improve our understanding of how individual pathways function. By expanding individual sequence datasets to populations, multiple species within a genus or even larger taxonomic groups, pangenomics and pantranscriptomics are highly promising approaches to complement and improve the current toolkit for studying specialised metabolism in plants. Pangenomic approaches integrate genomic information from multiple individuals and thereby reduce reference bias while capturing a broader spectrum of diversity (Bayer et al., 2020; Golicz et al., 2020). Because a single linear reference genome sequence represents only one genotype and therefore captures only a fraction of the genomic diversity present across a species, genes or structural variants present only in certain cultivars, landraces or wild relatives can be overlooked in reference‐based analyses. While pangenomics is a powerful tool to reveal the wider genetic repertoire of a species, it is static and does not allow any conclusions about the activity of these genes under different conditions or in different parts of the plant. Pantranscriptomics provides these functional insights into the activity of different genes and is therefore much more informative with respect to the actual metabolic activities in a plant. Since the regulation of plant metabolism is often more flexible during evolution than the biosynthesis genes themselves (McConnachie et al., 2025; Zhou & Liu, 2022), pantranscriptomics is a particularly powerful tool for the discovery of lineage‐specific innovations.

Here, we review how pangenomic and pantranscriptomic resources can be harnessed for elucidating and studying specialised plant metabolism. The current state of research is presented with recent examples of successful projects. Furthermore, we list and discuss databases that compile pangenomic and pantranscriptomic resources. Lastly, we discuss current trends and future developments in the field. Through this, our review highlights how pangenomics and pantranscriptomics help to shape the field of specialised metabolism in plants.

## PANGENOMICS

### Definition of pangenomics

A pangenome is the complete, non‐redundant set of genes and genomic sequences present within a defined biological group. The computational framework used to represent genomic variation across all members of that group is referred to as a pangenome sequence or a pangenome graph, depending on the underlying representation. The computational representation of pangenomes along with the analysis and interpretation collectively constitute the field of pangenomics (Tettelin et al., 2005).

Although most commonly constructed at the species level, pangenome sequences can also be generated for genera, wider phylogenetic lineages or even ecological communities. Conceptually, a pangenome is often partitioned into a core genome, comprising genes and intergenic regions shared by all sampled individuals and an accessory genome (also termed as dispensable or variable genome), consisting of genes present in only a subset of sampled individuals (Tettelin et al., 2005). The boundary between core and accessory genome is, however, influenced by sampling design and technical factors: First, pangenome composition depends on the number and phylogenetic breadth of individuals included. Broader sampling increases the possibility of discovering rare variants and accessory genes. Second, incomplete or fragmented assemblies can artificially inflate estimates of gene absence. Third, inconsistent annotation pipelines in individual genome sequences would lead to potential misclassification (Gordon et al., 2017). For these reasons, the dispensable genome frequently employs a more nuanced classification, distinguishing soft‐core genes (present in most genomes), shell genes (less frequent than soft‐core) and cloud genes (limited to only a few individuals) (Gordon et al., 2017) (Figure 1a).

**Figure 1:**
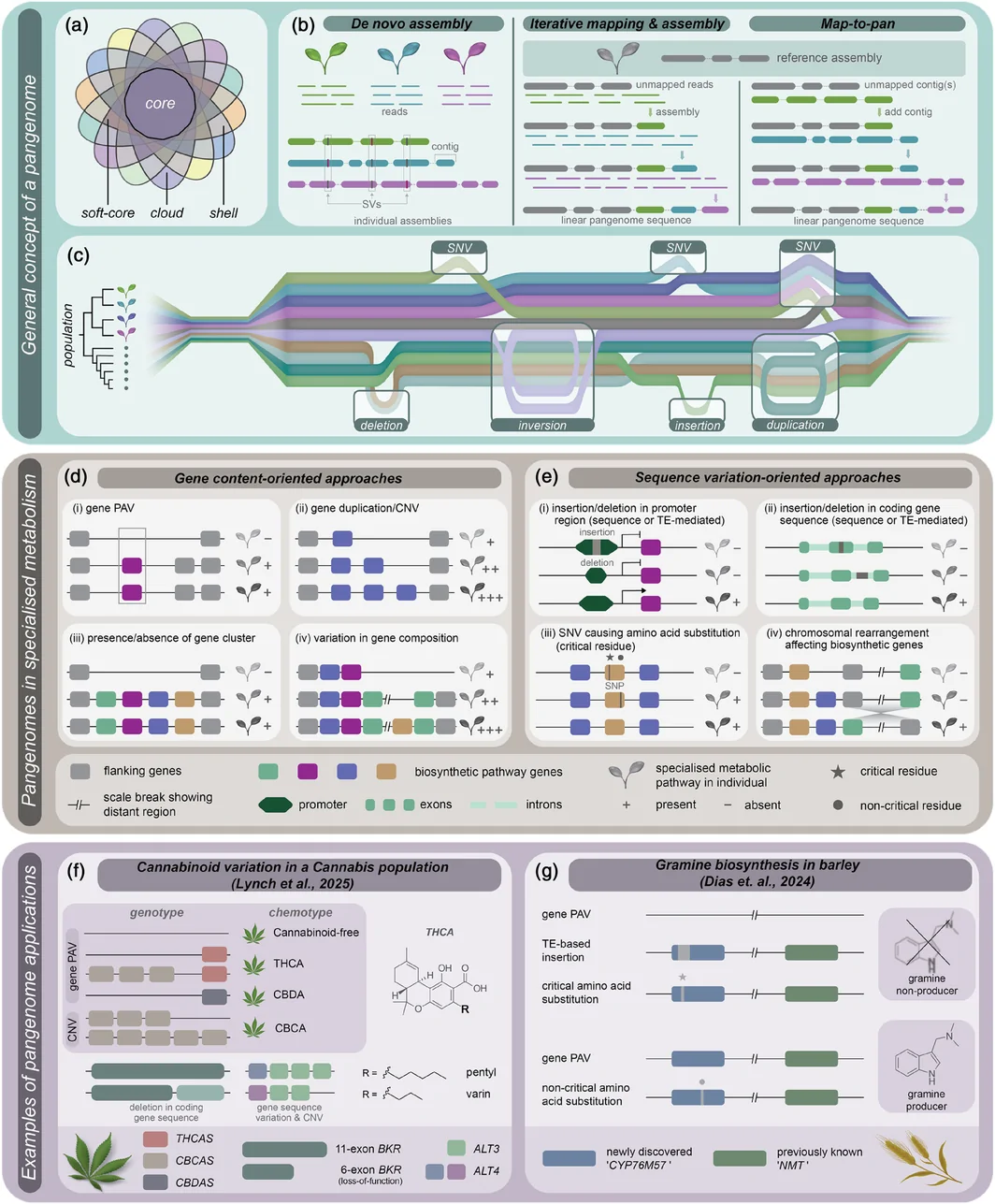
Pangenomes harbour multilayered information relevant for the investigation of plant specialised metabolism. (a) The concept of a pangenome: Each colour represents a genome implemented in the pangenome. Genes are classified as core, soft‐core, shell or cloud components of the pangenome based on appearance throughout the genomes. (b) Three main techniques to generate a linear pangenome as described in the text. (c) Representation of a pangenome graph. (d, e) Presence (+) and absence (−) of metabolic features in plants can be studied through gene content‐oriented approaches or through sequence variation‐oriented approaches. (f) PAVs and CNVs in THCA, CBDA and CBCA synthase genes lead to varying cannabinoid chemotypes in *Cannabis sativa*. The THCA structure is shown here as a representative; the presence of varin cannabinoids could be related to SVs in *BKR* and *ALT* genes (Lynch et al., 2025). (g) PAVs of and SVs in the gene encoding CYP76M57 in *Hordeum vulgare* explain why some barley accessions produce the alkaloid gramine and some do not (Dias et al., 2024). ALT, acyl‐lipid thioesterase; BKR, β‐keto acyl carrier protein reductase; CBCA, cannabichromenic acid; CBDA, cannabidiolic acid; CNV, copy‐number variation; CYP, cytochrome P450 monooxygenase; NMT, *N*‐methyltransferase; PAV, presence–absence variation; SNP, single nucleotide polymorphism; SNV, single nucleotide variation; SV, structural variation; TE, transposable element; THCA, tetrahydrocannabinolic acid.

The pangenome concept was first introduced in bacteria two decades ago (Tettelin et al., 2005), where extensive gene‐content variation among *Streptococcus agalactiae* strains was identified. Applications of pangenomics to plants followed substantially later, largely because routine plant genome sequence assembly was still a challenge owing to large genome sizes, high repeat content, frequent polyploidy and high heterozygosity, which contribute to the structural complexity of plant genomes. Progress in long‐read sequencing, scaffolding technologies, optical mapping and assembly algorithms has since transformed this landscape, making routine construction of multiple high‐quality plant genome sequences and therefore reliable plant pangenome studies increasingly feasible (de Oliveira et al., 2026).

The first plant pangenome sequence was reported for the wild progenitor of domesticated soybean (Li et al., 2014), where genome sequences of seven distinct accessions of *Glycine soja* were assembled *de novo*. This study identified structural variations in core and dispensable gene sets and offered candidate genes with potential agronomic value. Since then, plant pangenomics has been applied to numerous crops and model species, including rice (Guo, Li, et al., 2025), maize (Yang et al., 2025), wheat (Jiao et al., 2025), tomato (Shi et al., 2026), mustard (Ma et al., 2026), barley (Jayakodi et al., 2024), potato (Cheng et al., 2025), grapevine (Liu et al., 2024), lettuce (Cao et al., 2025) and *Arabidopsis thaliana* (Obinu et al., 2025). More recently, pangenomic studies have expanded beyond major crops and model systems, now including bryophytes (Beaulieu et al., 2025), rhododendron (Zhou et al., 2026), tulip tree (Wu et al., 2025) and other trees (Blanchard et al., 2026; Liang et al., 2025; Shi et al., 2024).

Several strategies are currently used to construct plant pangenome sequences (Figure 1b). The most conceptually straightforward one is the *de novo* assembly‐based approach, in which multiple genome sequences are independently assembled, followed by comparative analysis to detect gene presence–absence variation (PAV) and structural variants (SVs). For instance, the rice pangenome investigated by Zhao *et al*. in 2018 using short‐read sequencing resulted in the assembly of 66 divergent accessions of cultivated and wild rice and identified 23 million SVs across the rice pangenome (Zhao et al., 2018). Another rice pangenome study in 2022 used long‐read sequencing to assemble the genome sequences of 111 accessions of wild and cultivated rice, identifying 879 Mb and 19 000 novel sequences and genes, respectively (Zhang et al., 2022). A third rice pangenome sequence, published in 2023 was based on *de novo* assembly of 74 wild and cultivated rice accessions and identified a 22 Mb putative introgression region in cultivated rice accessions (Wu, Xie, et al., 2023). The *de novo* strategy typically provides high resolution for structural variant discovery and novel sequence detection; however, it is computationally demanding and can be expensive for large or polyploid genomes that require high sequencing coverage.

A second class of methods uses a reference‐based iterative strategy, in which sequencing reads are mapped to an existing reference genome sequence. The approach can be further divided into the ‘iterative mapping and assembly’ method and the ‘map‐to‐pan’ method. In the ‘iterative mapping and assembly’ approach, reads from each individual are iteratively aligned to a reference genome sequence, after which unmapped reads are assembled and the resulting contigs are incorporated into the reference, thereby generating a linear reference pangenome sequence (Hu et al., 2020). Because this strategy usually relies on short reads and lower sequencing coverage, it is comparatively cheaper, but less informative. Among other applications, this method was recently employed to construct a lettuce ‘super‐pangenome’, a pangenome sequence representing multiple species within the genus *Lactuca* (van Workum et al., 2024). In the ‘map‐to‐pan’ approach, reads are first *de novo* assembled and the resulting contigs are then aligned to a reference genome sequence. Unaligned contigs are then added to the reference, generating a linear reference pangenome sequence. Owing to its relatively simple workflow and efficient output, this strategy has been widely used in crop pangenomes including rice (Wang et al., 2018), tomato (Gao et al., 2019), cotton (He et al., 2024; Li et al., 2021) and others (Golicz et al., 2016; Wu, Sun, et al., 2023).

A third and increasingly applied approach is the pangenome graph representation (Figure 1c). Unlike the traditional linear format, pangenome graphs represent shared sequences as common paths, while complex rearrangements or alternative alleles at the same locus are represented as branching paths within a graph structure. This architecture captures complex variation within a single unified compact structure. Different graph models can be used to represent pangenome graphs including variation (or sequence) graphs, de Bruijn graphs and cactus graphs. Variation graphs are currently the most widely used graph model in plant pangenomics. They are typically constructed from whole‐genome sequence alignments or variant call format (VCF) files, with each base represented as a node, and edges connecting adjacent nodes denote the relationships of the sequences across genomes. Variants among individuals are shown as ‘bubbles’, allowing specialised tools to detect them with greater sensitivity than for linear pangenome sequences. Variation graphs can be generated using reference‐based approaches, for instance, using Minigraph‐Cactus (Hickey et al., 2024) or through reference‐free methods like Pangenome Graph Builder (PGGB) (Garrison et al., 2024). De Bruijn graphs constitute a reference‐free and alignment‐free alternative in which genome sequences are decomposed into overlapping k‐mers. Identical k‐mers are merged into shared nodes, whereas sequence differences generate alternative graph structures (Iqbal et al., 2012). Although highly efficient for representing sequence variation, de Bruijn graphs are less suitable for capturing large structural variants, often collapse repetitive regions and can obscure long‐range haplotype information because sequences are fragmented into short k‐mer units. Cactus graphs, on the other hand, are based on whole‐genome sequence alignments, which encode nested structural variation in hierarchical graph structures.

Most pangenome graphs are stored using the Graphical Fragment Assembly (GFA) format. Compared with linear pangenome representations, graph‐based approaches provide a richer catalogue of genomic variation, improved representation of structural rearrangements and path‐aware genomic coordinates. An additional advantage is their extensibility, as newly generated assemblies can be incorporated into existing graphs without reconstructing the entire pangenome (Denti et al., 2026). Pangenome graphs have been widely applied to plants, including *Arabidopsis thaliana* (Igolkina et al., 2025), sugarcane (Huang et al., 2026), potato (Zhu et al., 2025) and eggplant (Gaccione et al., 2025) and are expected to become a dominant framework for future pangenomics studies. Recent advances now enable haplotype‐resolved (Cheng et al., 2025; Sun et al., 2025) and telomere‐to‐telomere assemblies (Olbrich et al., 2025; Zhang, Zheng, & Chen, 2026) to be integrated into pangenome graphs, hence improving the resolution of allelic diversity in heterozygous and polyploid species.

The growing availability of pangenome sequences has also accelerated the development of dedicated tools and software for pangenome analysis, annotation pipelines, gene presence–absence analysis and SV detection. A comprehensive survey of these resources is beyond the scope of this review, but they have been reviewed in detail elsewhere (Du et al., 2025; Heuermann et al., 2026; Hu et al., 2024; Ruperao et al., 2025).

### Pangenomics‐based investigation of specialised metabolism

The ability to construct comprehensive pangenome sequences enables new applications in the field of specialised metabolism in plants. Generally, the rapidly improving availability of high‐quality plant genome assemblies has accelerated the discovery of specialised metabolic pathways in plants. Genome mining and comparative genomics have therefore become powerful strategies for elucidating specialised metabolism in plants (Jaczkowski et al., 2026; Polturak et al., 2022; Smit & Lichman, 2022; Swamidatta & Lichman, 2024). However, analyses based on only one or a few reference genome sequences are often insufficient to capture the extensive genomic variation underlying metabolic traits. The evolution of biosynthetic pathways is driven by rapid, diverse and often lineage‐specific genomic processes such as gene and genome duplications, many of which are absent in a single‐reference genome. Pangenomic approaches extend these capabilities by integrating multiple genomes within a species or lineage, thereby capturing both gene‐content and sequence‐level variation associated with metabolic diversity. Importantly, metabolic gain or loss is rarely attributable to a single variant class; rather, it often reflects the combined effects of gene‐content changes and regulatory variation.

In this section, we highlight representative examples in which pangenomics has accelerated the elucidation and study of specialised metabolic pathways in plants. These examples build on two broad main strategies: gene content‐oriented approaches, in which known or candidate genes are compared across pangenome members, and sequence variation‐oriented approaches, in which structural genomic features are identified and linked to differences in specialised metabolic pathways (Figure 1d,e).

#### Gene‐content variation as a driver of metabolic diversity

Variation in gene content is one of the most direct mechanisms that generate metabolic diversity. PAV, copy‐number variation (CNV), tandem duplication, biosynthetic gene cluster presence or lineage‐specific gene gains or losses can all alter pathway composition and metabolic output (Figure 1d).

An archetypical example comes from *Cannabis sativa*, where a pangenome comprising 193 genome sequences of 144 accessions revealed extensive variation in cannabinoid biosynthetic genes (Lynch et al., 2025). Some of the accessions contained only either a single functional *THCAS* or a *CBDAS* gene, encoding enzymes responsible for tetrahydrocannabinolic acid and cannabidiolic acid biosynthesis, respectively, while others contained both. In addition, both PAV and CNV were observed for *CBCAS*, the gene product of which catalyses the formation of cannabichromenic acid. The gene‐content differences of *THCAS*, *CBDAS* and *CBCAS* largely determine cannabis chemotypes (Lynch et al., 2025) (Figure 1f).

Pangenome analyses have also proven valuable for resolving incomplete pathways by correlating gene PAV with metabolic phenotypes. In barley, (Dias et al., 2024) used an existing barley pangenome sequence (Jayakodi et al., 2020) to investigate the biosynthesis of the defence indole alkaloid gramine. Comparative analysis between gramine‐producing and non‐producing accessions identified PAV in *CYP76M57*, a cytochrome P450 gene absent from non‐producing lines (Dias et al., 2024). Functional characterisation showed that CYP76M57 catalyses the conversion of tryptophan to aminomethylindole (AMI), the key precursor of gramine, thereby completing the gramine biosynthetic pathway in barley (Dias et al., 2024; Ishikawa et al., 2024) (Figure 1g).

Gene‐content analyses are powerful for investigating biosynthetic gene clusters and expanded gene families. In maize, integration of pangenomic and multiomics datasets enabled the identification of ten genes organised into three zealexin biosynthetic gene clusters, encoding four sesquiterpene synthases and six cytochrome P450 enzymes involved in pathogen defence (Ding et al., 2020). Likewise, a pangenome study of tomato including 61 accessions identified 2199 *O*‐methyltransferases (OMT) genes and uncovered tandemly duplicated OMT clusters associated with the biosynthesis of *O*‐methylated flavonoids such as kaempferide or chrysoeriol (Wang et al., 2026). Similarly, a gene‐based super‐pangenome of Polygonaceae species revealed substantial CNVs in flavonoid and anthraquinone biosynthetic genes across eleven genomes (Liu, Li, et al., 2025). Most interestingly, five tandemly duplicated copies of a rhamnosyltransferase gene involved in rutin biosynthesis were identified in *Fagopyrum esculentum* and *F. tataricum*, two species known for their high rutin content (Liu, Li, et al., 2025). Three additional tandem duplicated copies of genes involved in rutin degradation were also observed (Liu, Li, et al., 2025). These duplications were absent from related species with lower rutin content, highlighting that gene family expansion and contraction might have contributed to metabolic diversification in Polygonaceae (Liu, Li, et al., 2025).

#### Sequence variation and regulatory evolution of specialised metabolism

While gene‐content variation determines which biosynthetic genes are present, sequence variation influences how these genes function and are regulated. Structural variants (SVs), insertions and deletions in promoter regions, transposable element activity, coding‐sequence mutations and large chromosomal rearrangements can all affect metabolic phenotypes of biosynthetic pathways (Figure 1e).

In the same cannabis study mentioned above, Lynch *et al*. identified sequence variation in acyl‐lipid thioesterase (ALT) genes associated with differences in fatty acid chain length and production of rarer varin cannabinoids, which possess shorter fatty acid‐derived alkyl side chains compared to more common cannabinoids (Lynch et al., 2025). Additionally, they identified a 2‐bp deletion in the β‐keto acyl carrier protein reductase (BKR) gene that converted an eleven‐exon gene into a truncated six‐exon form resulting in a gene product that lacks key catalytic residues. This loss‐of‐function allele could lead to an increase in the butyryl‐acyl carrier protein pool that the *ALT* gene products act on, resulting in higher varin‐cannabinoid contents (Figure 1f) (Lynch et al., 2025).

Many (specialised) metabolic traits are shaped by structural variation in regulatory regions. Using a pangenome graph of 18 *Citrus* and *Citrus*‐related accessions, Huang *et al*. showed that genes exhibiting differential expression between lineages were frequently associated with promoter SVs (Huang et al., 2023). Using a multiomics approach, the authors identified a 1.2 kb promoter insertion affecting the transcription factor PH4, which was found to regulate vacuolar acidification and citric acid accumulation. This structural variant explained major differences in fruit acidity between *Citrus* and related genera (Huang et al., 2023). Similar patterns have also been observed in other plants. In tomato, a pangenome study encompassing 725 accessions uncovered a ~4 kb promoter substitution in *TomLoxC* (Solyc01g006540), encoding a 13‐lipoxygenase involved in the synthesis of lipid‐derived volatiles which contribute to desirable tomato flavour (Gao et al., 2019). Further characterisation of TomLoxC revealed its role in apocarotenoid production (Gao et al., 2019). In tea (*Camellia*), comparison of wild and domesticated accessions using a pangenome graph revealed a 192 bp insertion in the promoter of *ANS3* associated with increased anthocyanins in wild populations (Tao et al., 2025).

Pangenomics can also provide insights into the evolutionary mechanisms shaping metabolic pathways. In *Arabidopsis thaliana*, analyses of 22 *de novo* genome assemblies suggested that the thalianol biosynthetic gene cluster evolved through a series of chromosomal inversions and rearrangements that organised pathway genes into a physically linked cluster (Liu, Cheema, et al., 2020). Finally, sequence‐level variation within coding and regulatory regions can directly affect pathway activity. A *Sorghum* pangenome study identified sequence variants associated with differences in dhurrin accumulation, including an alanine‐to‐valine missense mutation in the biosynthetic gene *CYP79A1* and a 2‐bp deletion within a regulatory region predicted to disrupt binding sites for the abscisic acid‐responsive transcription factors ABF3 and ABF4 (Morris et al., 2026). These variants were predicted to increase the binding affinity of CYP79A1 for its substrate, tyrosine and alter transcriptional regulation of the biosynthetic pathway, respectively, providing a mechanistic explanation of natural variation in cyanogenic glycoside content among sorghum accessions (Morris et al., 2026).

Overall, pangenome sequences provide an excellent resource to study the genetic mechanisms underlying specialised metabolism. By simultaneously capturing gene‐content variation, regulatory divergence and structural genome dynamics, pangenomes enable researchers to connect genomic diversity with metabolic phenotypes and accelerate both pathway discovery and evolutionary studies. In practice, gene‐content and sequence variation approaches are often complementary, and their integration with transcriptomic, metabolomic and other functional datasets represents a powerful strategy for elucidating plant specialised metabolism.

## PANTRANSCRIPTOMICS

### Definition of pantranscriptomics

From pangenomics, it becomes evident that individual species harbour a core and a dispensable gene set, with some genes being located on a spectrum between these extremes. The latter mainly contributes to the PAV of genes observed at the individual level (Ma et al., 2019). However, not only the static gene content but also the dynamic expression levels of genes across individuals account for phenotypic differences (Sibbesen et al., 2023). A pantranscriptome can thus be defined as a population‐level collection of transcripts and their abundances under specific conditions across individuals or haplotypes at a given time point (Figure 2a) (Sibbesen et al., 2023). Obtaining a pantranscriptome becomes important to understand variation at the transcriptional and post‐transcriptional levels in different individuals or cultivars within the same species (Guo, Schreiber, et al., 2025). To put it simply, a pantranscriptome is an atlas of abundance variation across transcripts in individuals and subgenomes of a species (Lichman et al., 2020). Regulatory and epigenetic elements add further nuance to PAVs, based not only on gene content but also on gene expression, which all directly influence the phenotype observed (Petek et al., 2020). This perspective is particularly important for studying specialised metabolism, as such pathways in plants are often sculpted by evolutionary phenomena such as polyploidisation and gene duplication, with far‐ranging impact on gene expression as well as evolutionary fates – subfunctionalisation, neofunctionalisation and pseudogenisation (Lichman et al., 2020; White et al., 2025). For instance, a pantranscriptome study of the polyploid *Brassica napus* accessions revealed unequal or asymmetric gene expression among the different subgenomes (An et al., 2019). This underscores the need for a pantranscriptome‐powered approach to improve functional analysis of plant genes for elucidating and studying specialised metabolic pathways. A notable practical limitation of such studies, however, is the need for clear knowledge or at least hypotheses regarding biosynthetically active tissues and developmental stages. These are critical to obtain a pantranscriptomics dataset that is sufficiently focused for specific questions in plant specialised metabolism.

**Figure 2:**
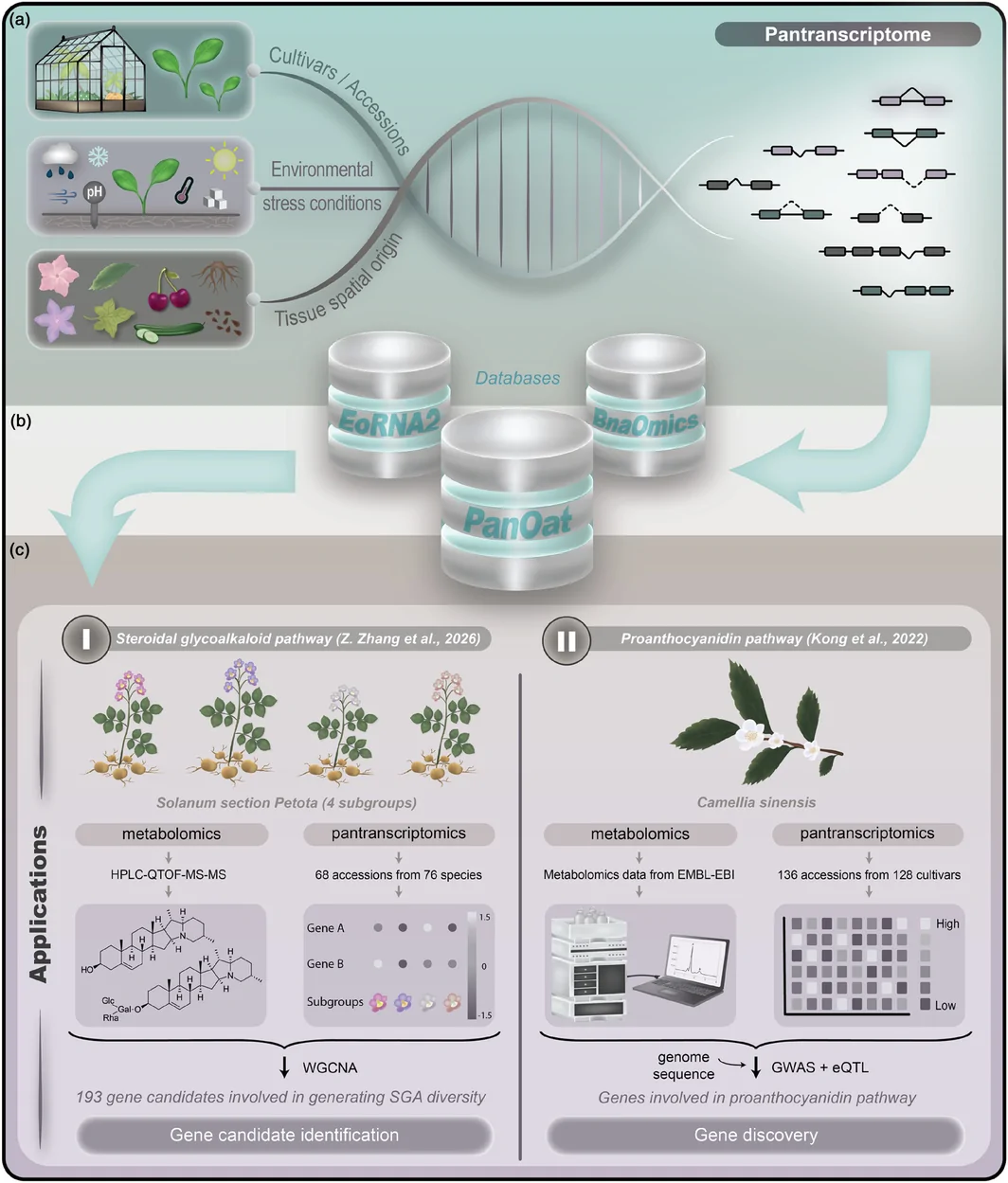
Pantranscriptomics‐based approaches for studying metabolic pathways in plants. (a) Pantranscriptomes representing the different variables and factors contributing to transcript abundances like accession differences, environmental conditions, spatial origin and biological events like alternative splicing. (b) Major pantranscriptomic databases (EoRNA2 (Milne et al., 2026), PanOat (Avni et al., 2026), BnaOmics (Cui et al., 2023)) are currently available. (c) Schematic workflow of exemplary studies leveraging pantranscriptomics to address various biological questions around plant specialised metabolism. (I) Application of pantranscriptomics and metabolomics to identify genes responsible for SGA diversity in plants of *Solanum* section *Petota*; gene expression profiles of the SGA biosynthesis genes across the different subgroups obtained via pantranscriptomics are represented by the heatmap with normalised FPKM values (Zhang, Wu, et al., 2026). (II) Integration of pantranscriptomics and metabolomics to identify genes involved in flavonoid biosynthesis in *Camellia sinensis* (Kong et al., 2022). EMBL‐EBI, European Molecular Biology Laboratory – European Bioinformatics Institute; eQTL, expression quantitative trait loci; FPKM, fragments per kilobase of transcript length per million reads; GWAS, Genome‐wide association study; HPLC‐QTOF‐MS–MS, high‐performance liquid chromatography‐quadrupole time‐of‐flight tandem mass spectrometry; SGA, steroidal glycoalkaloids; WGCNA, weighted gene co‐expression network analysis.

Continuous progress in RNA‐Seq technologies is transforming the plant transcriptomics landscape. While long‐read technologies such as PacBio isoform sequencing (Iso‐Seq), ONT‐based cDNA sequencing and direct RNA sequencing circumvent the need for error‐prone *de novo* transcriptome assembly, methods combining long‐read sequencing and short‐read Illumina‐based RNA‐seq data are routinely used for pantranscriptome construction (Chen et al., 2025; Guo, Schreiber, et al., 2025; Zhong et al., 2024). Guo and co‐workers used this combined approach to merge the short‐ and long‐read transcriptome assemblies by including only those transcripts from short reads that contained a new splice junction or revealed new gene loci (Guo, Schreiber, et al., 2025). That said, while there is no universal method to obtain a pantranscriptome, approaches can be broadly categorised into being pangenome sequence‐dependent or ‐independent (Table 1). A pangenome‐dependent pantranscriptome construction method was adopted by Guo and co‐workers (Guo, Schreiber, et al., 2025). It includes trimming and a quality check of the RNA‐Seq reads, mapping them to the respective individual reference genome sequences, obtaining the transcriptome assemblies and finally mapping the genotype‐specific reference transcripts against a pangenome (Avni et al., 2026; Guo, Schreiber, et al., 2025). The pangenome‐free approach was adopted by Petek and co‐workers to obtain a pantranscriptome of potato cultivars (Petek et al., 2020). Their workflow also begins with a quality check and trimming step of RNA‐Seq reads but is followed by *de novo* transcriptome assembly instead of mapping to a reference genome sequence, removal of redundant sequences while retaining the longest transcripts and finally merging the transcripts from the different cultivars to generate the pantranscriptome assembly. Such pantranscriptome assemblies can then be further used for gene expression quantification and functional annotation to aid in gene function elucidation for pathway discovery. Computational tools like RPVG (Sibbesen et al., 2023) can facilitate such tasks. RPVG, a standalone tool offered along with the VG toolkit, can use the RNA‐Seq alignments performed against a pangenome graph and quantify the haplotype‐specific transcript abundance without the need to genotype the individual (Sibbesen et al., 2023). Such useful tools and computational techniques are helping to transfer developments from pangenomics over to pantranscriptomics, a scientific field with immense potential for bioinformatics methods and tool development.

**Table 1:** Comparison of the two major pantranscriptome construction approaches, pangenome sequence‐dependent and ‐independent

Approach	Input(s)	Output	Relative computational cost	Recommended use case
Pangenome sequence‐dependent	RNA‐Seq, Iso‐Seq and/ or direct RNA sequencing data per cultivar High‐quality reference genome assembly per cultivar Pangenome resource	Unified pantranscriptome linkable to structural genomic features	High	For research questions entailing connecting expression to structural genomic features High‐quality chromosome‐scale genome assemblies per cultivar/ accession are available Pantranscriptome requires to be genotype‐resolved
Pangenome sequence‐independent	RNA‐Seq, Iso‐Seq and/ or direct RNA sequencing data per cultivar	Combined pantranscriptome encompassing cultivar‐specific *de novo* transcriptome assemblies	Low to moderate	Research focus tends more towards novel isoform discovery or expression‐based cultivar‐specific marker identification Lack of high‐quality genome assemblies per cultivar, especially for non‐model species

### Examples of pantranscriptomics studies of plant specialised metabolism

Pantranscriptomic approaches have been used for a variety of applications in plant sciences; this section mainly focuses on case studies related to plant biosynthetic pathways but also refers to selected examples from plant metabolism in general.

Steroidal glycoalkaloids (SGAs) are produced primarily by *Solanum* plants. They play a role in defence against pathogens and herbivores; on the other hand, accumulation of these compounds in edible tissues contributes to bitterness and toxicity to humans. Thus, it is important to develop crops with improved phytochemical profiles. The biosynthetic pathways leading to structurally diverse SGAs in the *Solanum* section *Petota*, which includes potato, have not been studied in detail yet. Zhang and co‐workers therefore adopted an integrated omics approach to unravel unknown biosynthetic players involved in SGA biosynthesis (Zhang, Wu, et al., 2026). They constructed a high‐quality pantranscriptome from 68 accessions belonging to 46 species of *Solanum* section *Petota* which helped them obtain ~5000 assembled sequences that were absent in the reference genome sequence, highlighting the usefulness of a pantranscriptome dataset. Next, metabolic profiling of SGAs from leaf samples of all the accessions led to the identification of 64 structurally distinct SGAs. Lastly, the pantranscriptome and metabolome datasets were integrated via weighted gene co‐expression network analysis (WGCNA) that helped find 193 gene candidates that might be involved in generating the diversity of SGAs (Figure 2c) (Zhang, Wu, et al., 2026).

The dried roots of *Salvia miltiorrhiza* are of substantial pharmacological interest due to their accumulation of bioactive compounds. Tanshinones are diterpenoids which are part of the active compounds in the root periderm of this plant. However, tanshinone accumulation is low in the roots and varies according to the geographical location of the plant, limiting its utility in industry. This motivated a research team to unravel the core regulatory triggers impacting tanshinone biosynthesis (Yao, Liao, et al., 2026). Treatments with exogenous hormones revealed methyl jasmonate to be a positive enhancer signal increasing tanshinone biosynthesis (Yao, Liao, et al., 2026). To further understand the regulatory mechanisms behind this effect, the authors constructed a pantranscriptome dataset of *S. miltiorrhiza* with over 50 publicly available samples encompassing different tissue types, ecotypes, developmental stages and treatments. They constructed a hormone treatment–tanshinone correlation network using the pantranscriptome dataset and followed up with a gene set enrichment analysis. Based on previous reports on MYC transcription factors (TFs) playing a crucial role in jasmonate signalling, the authors focused on the expression levels of MYC TFs across different treatments and locations in the pantranscriptome dataset‐derived expression matrix (Yao, Liao, et al., 2026). A differential gene expression analysis of the MYC TFs across low and high tanshinone‐accumulating ecotypes led to the discovery of a new TF, SmMYC3. Analysis of overexpression and silenced lines helped functionally validate the role of this new TF; biochemical assays showed that it binds to the G‐box element motif in the promoter sequence of key tanshinone biosynthesis genes (Yao, Liao, et al., 2026).

Beyond such experimentally supported examples, several studies have used pantranscriptomic approaches for candidate gene discovery based mainly on *in silico* analyses. In the tea plant *Camellia sinensis*, pantranscriptomics has been directly applied to investigate the genetic basis of specialised metabolism: Kong *et al*. constructed a population‐scale pantranscriptome assembly and integrated it with metabolite profiling and association analyses to identify genes associated with variation in key specialised metabolites such as flavan‐3‐ols (including both monomeric catechins and polymeric proanthocyanidins) (Kong et al., 2022). By combining pantranscriptomic expression data with genome‐wide association studies (GWAS) and expression quantitative trait locus (eQTL) mapping, the study was able to link genotype‐specific transcripts and regulatory variation to metabolite accumulation patterns across tea accessions. Notably, candidate genes involved in flavonoid biosynthesis, such as a putative orthologue of *ANS*, were associated with proanthocyanidin accumulation, while variation in catechin composition was linked to genes encoding flavonoid biosynthetic enzymes, including the flavonoid hydroxylase F3'5'H (Figure 2c) (Kong et al., 2022).

In contrast to studies directly linking pantranscriptomics to specialised metabolic traits, several analyses have focused on characterising the extent and functional relevance of transcriptomic variation itself. In maize, (Jin et al., 2016) demonstrated that expression presence–absence variation (ePAV) is widespread and functionally relevant, linking transcript‐level variation to regulatory divergence and metabolic trait variation. In parallel, a recent study in hexaploid wheat revealed a substantial fraction of cultivar‐specific transcripts, extensive transcript isoform diversity and widespread variation in homoeologue expression bias across subgenomes, highlighting pronounced transcriptional divergence between genotypes (White et al., 2025).

A major part of applying pantranscriptomics besides biosynthetic pathway elucidation is to analyse and improve crop breeding (Admas et al., 2025). One target for improvement can be abiotic or biotic stress responses and the connected regulatory pathways, as they play key roles in plant adaptation to environmental conditions. Biosynthetic pathways leading to specialised metabolites are often regulated at the transcriptional level. This regulation is frequently tied to biotic or abiotic stress responses in plants (Cao et al., 2024). Hence, techniques to study these regulatory elements might also find useful applications in specialised metabolism research. In plant breeding, one major focus of the application of pantranscriptomics is the investigation of non‐coding RNAs (ncRNAs). They are represented in plant transcriptomes and are reported to regulate gene expression (Cao et al., 2024). Medina *et al*. used a pantranscriptome analysis focused on long non‐coding RNAs (lncRNAs) and circular RNAs (circRNAs) for the investigation of salt and drought tolerance in alfalfa (*Medicago sativa*) (Medina et al., 2021). They generated a pantranscriptome of 21 samples including various tissues from three cultivars with different levels of salt or drought stress tolerance grown under salt‐, drought‐ or control conditions. They detected almost seven times more transcripts than reported in the alfalfa reference genome sequence, while uncovering nine new transcription factor families. The authors found that lncRNAs regulated the expression of adjacent genes and discovered enriched lncRNAs and circRNAs targeting genes encoding enzymes with UDP‐glycosyltransferase and oxidoreductase activity (Medina et al., 2021). In a weighted gene correlation network, the authors further investigated regulatory models to connect expression and traits based on gene expression in the respective cultivars. This led them to the central hub gene *G33123.7*, encoding a nerol synthase (producing an acyclic monoterpene alcohol) (Medina et al., 2021). The authors also found a MYB transcription factor regulating this nerol synthase gene among other genes (Medina et al., 2021). Besides ncRNAs, regulatory mechanisms can be mediated by alternative splicing events. In a study on cold stress response in *Oryza sativa*, the authors established a pantranscriptome including eleven samples from three rice cultivars with different cold tolerance levels (Zhong et al., 2024). They found that transposable elements were the main drivers for splice variants that determined the cold tolerance of the rice cultivars (Zhong et al., 2024). Hierarchical clustering of differentially expressed alternatively spliced genes brought forth a cluster that could be related to trehalose metabolism previously reported to contribute to cold stress tolerance (Zhong et al., 2024). The authors further found the splicing factor *OsRS2Z38*, encoding one of the two serine/arginine‐rich proteins that was upregulated during cold response in tolerant cultivars (Zhong et al., 2024).

Lastly, the concepts of presence–absence variants of all members of a gene family can be analysed across species using pantranscriptomics and pangenomics. To investigate the evolution of the bHLH TFs in barley, Tong *et al*. used the pangenomic nomenclature and identified core and dispensable members of the bHLH family in barley (Tettelin et al., 2005; Tong et al., 2025). The basis for the investigation was a previously established barley pangenome sequence (Jayakodi et al., 2020), from which the authors identified all members of the bHLH family using BITACORA, which is a tool for precise gene family annotation (Vizueta et al., 2020). The analysis included genes that were missed even in the annotated pangenome representation (Tong et al., 2025). This underlines the necessity of specific annotation for covering the whole diversity of a gene family in a species. This is especially true for the members of the bHLH family which were only present in some accessions and were associated with the cloud. There, the authors found 7 out of 20 genes that were not annotated in the pangenome representation (Tong et al., 2025). Further, they used a pantranscriptome (Guo, Schreiber, et al., 2025) to characterise transcriptional profiles and found that expression profiles of bHLH genes were not correlated with the pangenome attribution of the core or dispensable gene set (Tong et al., 2025). The expansion of a gene family is often accompanied by the accumulation of new traits based on newly gained enzymatic functions. As such traits are frequently connected to biosynthetic pathways, a combination of pangenomics and pantranscriptomics can be exploited to discover the routes to specialised metabolites.

## DATABASES

### Pangenomics databases

As the number of plant pangenome sequences has grown rapidly over the past decade, with numerous high‐quality pangenome sequences available now, it has become increasingly important to develop database resources to facilitate public access to this data. In most studies, only the individual genome assemblies and annotations are deposited in publicly available repositories. This limits the reusability of pangenomic datasets.

Multiple dedicated databases have been developed for individual species (Goodstein et al., 2012; Van Bel et al., 2022). The ‘Citrus Pan‐Genome to Breeding Database’ (CPBD) hosts genome assemblies and annotations for 23 accessions from the citrus family, Rutaceae, together with gene expression, structural variation, DNA methylome and collinearity blocks (Liu et al., 2022). However, the pangenome representation itself is not available. Rice research benefits from dedicated pangenome resources. The Rice Pangenome Browser (RPAN) provides a linear rice pangenome sequence constructed from 3010 genome sequences together with gene annotations, PAVs, expression profiles and an interactive browser for exploring the pangenome (Sun et al., 2017). The Rice Super Pangenome Information Resource Database (RiceSuperPIRdb) hosts a pangenome graph built from 251 rice accessions which can be explored interactively and downloaded alongside all individual genome assemblies (Shang et al., 2022). Other species‐specific resources include PanBARLEX for barley (Jayakodi et al., 2024), which enables interactive exploration of the barley pangenome derived from 76 diverse genotypes, but only allows downloading the protein‐coding gene annotations of the pangenome and not the complete pangenome representation. The ‘*Brassica napus* pan‐genome information resource’ (BnPIR) provides access to the rapeseed pangenome sequence together with variation data (Song et al., 2021). BnPIR serves as the genomic data source for the *Brassica napus* multiomics information resource (BnIR) (Yang et al., 2023), which integrates transcriptomics and metabolomics datasets. The Joint Genome Institute (JGI), through Phytozome (Goodstein et al., 2012), also hosts several species‐specific pangenome projects, namely BrachyPan (*Brachypodium distachyon*) (Gordon et al., 2017), CowpeaPan (*Vigna unguiculata*) (Liang et al., 2024), PennyPan (*Thlaspi arvense*) (Bird et al., 2026) and SorghumPan (*Sorghum bicolor*) (Morris et al., 2026). These have been developed to provide access to pangenome sequences and pangene sets for *Brachypodium distachyon* and *Sorghum bicolor* and individual genome assemblies, annotations and analysis tools for all.

In recent years, lineage‐specific pangenome resources that extend beyond single‐species scale have also been developed. A Solanaceae super‐pangenome from 30 accessions can be investigated using the Solanaceae Pangenome database (SolPGD), a platform for comparative genomics (Zhang et al., 2025). The Asteraceae Multi‐Omics Information Resource (AMIR) hosts genome assemblies and annotations together with additional omics datasets for 74 species, of which 43 were selected to represent a robust Asteraceae super‐pangenome (Liu, Luo, et al., 2025). Bryogenomes provides access to genome assemblies and annotations from 123 bryophyte species with integrated search tools like BLAST (Dong et al., 2025). While all three of the databases are valuable resources for comparative analyses, none of them feature a linear or graph pangenome representation. GreenPhylDB v5 provides homologous gene family analysis across 19 pangenome and 27 reference genome sequences (Guignon et al., 2021). It also provides access to pangenes for these 19 species but not the complete pangenome representation (Guignon et al., 2021). Gramene Plants provides dedicated pangenome portals for maize, rice, grapevine and sorghum (Olson et al., 2026). These portals integrate genome assemblies with gene annotations, genetic variants and expression datasets while providing user‐friendly browsing and visualisation tools (Olson et al., 2026). However, downloadable pangenome representations are not provided. GrainGenes hosts pangenome resources for several small‐grain cereals, including wheat, barley, oat and rye (Yao, Blake, et al., 2026). The platform offers pangenome BLAST searches and comparative micro‐ and macro‐synteny visualisation between assemblies. Similar to many other databases, integrated pangenome representations themselves are not available for download. Finally, one of the most comprehensive resources currently available is PlantPan, which provides standardised pangenome analyses across 12 species using more than 200 genome assemblies (Jiang et al., 2025). The database includes both gene‐centric and sequence‐centric pangenome representations, data on structural variation and synteny, gene clusters and functional annotations generated using a consistent analysis pipeline. Importantly, pangenome graphs for most included species are available for download, making PlantPan one of the few resources supporting direct reuse of complete pangenome datasets.

### Pantranscriptome databases

In contrast to pangenomes, only few repositories and databases have been developed for pantranscriptome studies in plants that can be used for analysing gene expression and function (Figure 2b). Organising pantranscriptome datasets generated in studies into dedicated repositories can simplify downstream expression data analysis and visualisation for biologists while conforming to the FAIR (Findable, Accessible, Interoperable, Reusable) data principles. BnaOmics is a comprehensive database that contains a pangenome of *Brassica napus* obtained from 18 cultivars but additionally facilitates in‐depth expression analysis in multiple tissues and populations, making it a valuable resource for breeding and gene discovery in *B. napus* (Cui et al., 2023). PanBaRT20 is a barley (*Hordeum vulgare*) pantranscriptome database encompassing five different tissue types from twenty different genotypes of barley (Guo, Schreiber, et al., 2025). The work also provides a gene expression atlas of the Morex cultivar that can be utilised for functional annotation (Milne et al., 2026). The datasets and MorexGeneAtlas underlying the PanBaRT20 study are also reflected in EoRNA, a gene expression database for barley released in 2021 (Milne et al., 2021). A more recent barley pantranscriptome repository is EoRNA2 (Milne et al., 2026), an updated release of the EoRNA database. Along with the improved EoRNA2 database, a completely automated Nextflow workflow is also available for users to obtain relevant RNA‐seq datasets and perform quality‐control and gene expression quantification seamlessly (Milne et al., 2026). More importantly, the database framework and infrastructure code have been generalised to improve their reusability for other species/taxa (Avni et al., 2026). Avni and co‐workers provided a valuable collection of chromosome‐level assemblies of thirty‐three oat genomes (Avni et al., 2026). They also provide an expression atlas across six tissue types in different development stages across twenty‐three oat lines that is useful to study the variation in gene expression due to gene loss and subgenome divergence in hexaploid oat. The expression matrices and orthology tables can be accessed for download from the PanOat section of the GrainGenes database (Avni et al., 2026). Together, these resources provide an important foundation for pantranscriptomic analyses and point to future opportunities for their application in specialised metabolism research. A summary of major plant pangenome and pantranscriptome databases along with their access links is provided in Table 2.

**Table 2:** Pangenome and pantranscriptome databases

Database name and access link	Taxa (Number of accessions)	References
Pangenome databases		
Asteraceae Multi‐omics Information Resource (AMIR), https://yanglab.hzau.edu.cn/AMIR/home/	Asteraceae (43)	Liu, Luo, et al. (2025)
Citrus Pan‐Genome to Breeding Database (CPBD), http://citrus.hzau.edu.cn/	*Citrus* (23)	Liu et al. (2022)
BnaOmics, https://bnaomics.ocri‐genomics.net/	*Brassica napus* (18)	Cui et al. (2023)
BrachyPan, https://phytozome‐next.jgi.doe.gov/brachypan	*Brachypodium distachyon* (54)	Gordon et al. (2017)
Brassica napus Pangenome Information Resource (BnPIR), http://cbi.hzau.edu.cn/bnapus/index.php	*Brassica napus* (1689)	Song et al. (2021)
Bryogenomes, http://www.bryogenomes.org/	Embryophyta (Bryophyta, Marchantiophyta, Anthocerotophyta) (123)	Dong et al. (2025)
CowpeaPan, https://phytozome‐next.jgi.doe.gov/cowpeapan/	*Vigna unguiculata* (7)	Liang et al. (2024)
GrainGenes, https://graingenes.org/GG3/pangenome	*Aegilops tauschii* (4), *Triticum monococcum* (2), *Hordeum vulgare* (10/76), *Avena* (3/3/33), *Triticum aestivum* (10/17)	Yao, Blake, et al. (2026)
Gramene Plants, https://www.gramene.org/pansites	*Zea mays* (56), *Oryza* (28), *Vitis* (29), *Sorghum bicolor* (85)	Olson et al. (2026)
GreenPhylDB v5, https://www.greenphyl.org/cgi‐bin/index.cgi	*Brachypodium distachyon* (54), *Brassica napus* (3), *Brassica oleracea* (3), *Brassica rapa* subsp. *rapa* (2), *Capsicum annuum* (3), *Cicer arietinum* (2), *Cocos nucifera* (2), *Cucumis sativus* (3), *Ipomoea trifida* (2), *Zea mays* (4), *Malus domestica* (3), *Medicago truncatula* (2), *Musa acuminata* (4), *Oryza sativa* (7), *Sorghum bicolor* (2), *Glycine max* (2), *Theobroma cacao* (2), *Triticum turgidum* (2), *Vitis vinifera* (3)	Guignon et al. (2021)
PanBARLEX, https://panbarlex.ipk‐gatersleben.de/	*Hordeum vulgare* (76)	Jayakodi et al. (2024)
PennyPan, https://phytozome‐next.jgi.doe.gov/pennypan/	*Thlaspi arvense* (7)	Bird et al. (2026)
PlantPan, https://ngdc.cncb.ac.cn/plantpan/	*Arabidopsis thaliana* (8), *Brassica napus* (5), *Brassica rapa* (13), *Zea mays* (5), *Solanum tuberosum* (8), *Raphanus sativus* (11), *Oryza sativa* (32), *Sorghum* (12), *Glycine* (29), *Fragaria ananassa* (5), *Solanum lycopersicum* (46), *Saccharum officinarum* (7)	Jiang et al. (2025)
Rice Super Pangenome Information Resource Database (RiceSuperPIRdb), http://www.ricesuperpir.com/	*Oryza* (*O. sativa* (202), *O. rufipogon* (28), *O. glaberrima* (11), *O. barthii* (10))	Shang et al. (2022)
Rice Pangenome Browser (RPAN), https://cgm.sjtu.edu.cn/3kricedb/	*Oryza sativa* (3010)	Sun et al. (2017)
Solanaceae Pangenome Database (SolPGD), http://www.bioinformaticslab.cn/SolPGD/#/	Solanaceae (30)	Zhang et al. (2025)
SorghumPan, https://phytozome‐next.jgi.doe.gov/sorghumpan/	*Sorghum bicolor* (32)	Morris et al. (2026)
Pantranscriptome databases		
EoRNA2, https://ics.hutton.ac.uk/eorna2/index.html	*Hordeum vulgare* (3 reference transcriptomes from more than 20 genotypes)	Milne et al. (2026)
PanBaRT20, https://ics.hutton.ac.uk/panbart20/index.html	*Hordeum vulgare* (5 tissues from 20 lines)	Guo, Schreiber, et al. (2025)
PanOat, https://graingenes.org/GG3/PanOat	*Avena sativa* (6 tissues from 23 lines)	Avni et al. (2026)

A continuously updated list of published studies that generated pangenome or pantranscriptome datasets is available as an online resource at https://github.com/PuckerLab/PlantGenomicsGuide/wiki.

As evident from Table 2, there is a noticeable difference between the number of pangenome and pantranscriptome databases. There is also a skew of database resources towards model or crop species (Lim et al., 2022; Panahi et al., 2026). Although expression data repositories exist for numerous plant species, they do not integrate genotype‐specific mapping and isoform‐level resolution unlike a pantranscriptomic resource. Major limitations for generating pantranscriptome resources of better scale and quality that will have to be resolved in the future are technical challenges associated with processing isoforms from a wide variety of samples and conditions (Panahi et al., 2026) and the associated difficulties in standardising data processing methodologies (Lim et al., 2022) and metadata information (Panahi et al., 2026).

## CONCLUSION OF TRENDS AND PROSPECTS

In the previous sections, we highlighted the most important developments in recent years regarding the use of pangenomics and pantranscriptomics for studying specialised metabolism in plants. Particularly noteworthy, pangenome graphs are increasingly regarded as the next major advance in plant science research since they comprehensively capture population‐scale sequence diversity. This is particularly evident in crop research, where characterising beneficial alleles and genes helps in devising informed breeding strategies (Guo, Li, et al., 2025; Liu et al., 2024; Liu, Du, et al., 2020; Zhou et al., 2022). However, for plant specialised metabolism, the application of pangenomics as a routine tool for the discovery of biosynthetic genes remains constrained by several practical challenges (Boxes 1 and 2).

Box 1 **Bullet‐Point Summary**

- Pangenomics captures the complete, non‐redundant set of genes and genomic sequences within a defined biological group such as a plant population.
- Likewise, pantranscriptomics provides a comprehensive set of transcripts and transcript abundances within a biological group, revealing which genes are active under the specific conditions at a given time point.
- Current examples of pangenomics in plant specialised metabolism are mainly focused on gene content and/or sequence variation.
- Pantranscriptomics examples have mainly investigated the regulation of biosynthetic networks.
- Various pangenomic and, to a lesser extent, pantranscriptomic databases provide access to datasets but mostly cover crop plants.

Box 2 **Open Questions**

- How can a complex pangenome be represented in a computationally efficient way?
- How can public datasets be integrated in pantranscriptomic studies aiming at the elucidation of biosynthetic networks?
- What are efficient strategies to integrate pangenome and pantranscriptome data for pathway elucidation and overcoming the functional annotation bottleneck in plants?
- How can technological developments in sequencing and machine learning be leveraged to navigate complex biosynthetic pathways with the help of pangenomics and pantranscriptomics?
- How can pangenomic and pantranscriptomic data generation and dissemination be standardised to account for the heterogeneity and biological complexity of the data and methods employed while ensuring long‐term accessibility and proper data provenance?

At the technical level, pangenome analysis remains computationally demanding, especially for plants. High‐performance computing infrastructure, substantial memory, long runtimes and extensive data storage are often required (Nie et al., 2026). In parallel, public infrastructure for long‐term storage, sharing and standardised dissemination of pangenomic data is still immature compared with the well‐established repositories for individual genome assemblies (Arita et al., 2021). Differences in methodologies further complicate the field, as different strategies for pangenome construction lead to substantial differences in estimates of core and dispensable genes, SVs and gene PAVs among studies. The next phase of pangenome research would benefit from community‐wide benchmarking studies and adoption of standardised metadata practices (Heuermann et al., 2026).

Comparable limitations are also evident for pantranscriptomics, although they mainly concern resource availability, functional integration and the restricted scope of current applications. Despite recent advances, applications of pantranscriptomics related to specialised metabolism remain scarce. While pantranscriptome approaches have proven effective in capturing transcript diversity beyond single‐reference frameworks, many current studies mainly use pantranscriptomics for transcript cataloguing, differential expression analyses or the investigation of population‐level variation (Guo, Schreiber, et al., 2025; Kong et al., 2022; White et al., 2025). Although these methods can support functional annotation and candidate gene discovery, their integration into pathway‐level analyses and experimental validation is often lacking. Recent work has  emphasised that pantranscriptomics captures transcriptional variation beyond gene presence/absence (Kanwal et al., 2025), yet its integration into downstream functional analyses remains underdeveloped. While several publicly available pantranscriptome resources exist for major food crops, the overall scope of such resources remains limited. There is also an enormous potential to integrate expression analysis with pantranscriptome resources or databases. An example is the set of gene expression tools provided as a part of the Bio‐Analytic resource for Plant Biology (Lim et al., 2022). A broader application of pantranscriptomics to study plant specialised metabolism would produce more diverse plant pantranscriptome resources. This can not only expedite plant pathway discovery by leveraging large‐scale comparative transcriptomics analyses but also provide valuable resources for other studies in plant biology.

Beyond these technical and resource‐related constraints, a central challenge is the biological complexity of specialised metabolism. In this context, the potential of pangenomics for pathway elucidation remains underexploited and situational. While pangenomes are highly effective for identifying biosynthetic gene clusters, tandem duplications and copy‐number variants, specialised metabolic traits are often controlled by multilayered regulatory systems, epigenetic states, tissue specificity or environmental responsiveness (Weng, 2014; Weng & Noel, 2012). In such cases, genomic variation alone may be insufficient to explain pathway activity or metabolite formation. Pangenomic analyses are most powerful when combined with functional multiomics datasets such as pantranscriptomics, metabolomics, expression quantitative trait loci and panepigenomics (Mishra et al., 2024). This is particularly important for specialised metabolism where a coordinated regulation of multiple genes is common (Mishra et al., 2024).

This need for integration also applies to transcriptomics‐based approaches. Several established transcriptomics‐based strategies for studying specialised metabolism have not yet been fully integrated into pantranscriptomic frameworks. One key example is comparative transcriptome‐metabolome profiling, which links differential gene expression to variation in metabolite accumulation in order to identify candidate pathway genes. While elements of this approach have been applied in tea (Kong et al., 2022), its systematic implementation in pantranscriptomics remains rare. Together, these limitations indicate that pangenomics and pantranscriptomics should not be viewed as isolated approaches, but as complementary layers that require functional and metabolomic integration to support pathway elucidation.

In addition to established transcriptomics strategies, recent methodological developments further suggest that pantranscriptomics could be extended beyond its current applications. Graph‐based and haplotype‐aware approaches such as those introduced by (Sibbesen et al., 2023) enable transcript quantification across complex genomic variation while reducing reference bias. Such approaches are particularly promising for specialised metabolism, where pathway genes are often associated with lineage‐specific metabolism and structurally variable genomic context (Smit & Lichman, 2022).

Furthermore, isoform‐resolved transcriptomics offers the potential to identify functionally distinct transcript variants, providing further insight into the contribution of transcript diversity to metabolic variation. Isoforms have also been found to play an important role in the regulation of specialised metabolic pathways such as flavonoid, theanine and caffeine biosynthesis in the tea plant (Qiao et al., 2019). Although alternative splicing has already been investigated in a pantranscriptomic context in rice and alfalfa (Medina et al., 2021; Zhong et al., 2024), isoform‐resolved pantranscriptomic approaches remain rarely applied but offer considerable potential for uncovering transcript variant‐specific contributions to pathway regulation and metabolite diversity (Lam et al., 2022).

However, the identification of genomic or transcriptomic variation alone does not necessarily provide functional insight. Although SVs and novel genes are frequently identified in pangenomics studies, the functional interpretation remains a major bottleneck. Translating these observations into biological insights requires additional annotation pipelines like protein structure prediction, knockout studies or co‐expression networks.

Despite these challenges, recent progress in pangenomics strongly suggests that it will become a lasting and central component of plant biology and specialised metabolism. Advances in haplotype phasing, telomere‐to‐telomere assembly and scalable graph algorithms are steadily reducing technical barriers (Shi et al., 2026; Zhang, Zheng, & Chen, 2026). At the same time, pangenomes are expanding beyond single‐species frameworks towards supra‐species scales, including genus and family level resources (Guo, Wang, et al., 2025; Khan et al., 2024; Raza et al., 2026; Zhang et al., 2024). Such higher‐order pangenomes are valuable for lineage‐specific, taxonomically patchy or repeatedly gained and lost metabolic pathways.

Beyond pangenomic resources, pantranscriptome resources could also strengthen comparative and cross‐species analyses. One underexplored opportunity is cross‐species function transfer, in which functional information from well‐characterised species is used to guide candidate gene identification in less‐studied species. While direct examples of cross‐species function transfer within plant pantranscriptomics are currently lacking, pantranscriptomic data can provide an additional layer for candidate function filtering. In this context, pangenomic analyses would define a broader set of orthologous candidate genes, while pantranscriptomic information could help assess which of these candidates show conserved or context‐matched expression patterns across accessions or related species. Another shared future direction is the use of artificial intelligence and machine learning. These technologies are expected to also accelerate pangenomic analyses by improving gene prediction, identifying and classifying structural variants, inferring gene presence–absence patterns and identifying candidate biosynthesis genes from complex graph structures. Recent developments further suggest that pangenomic resources can be used to train species‐specific DNA language models. For example, OryzaG3, a rice‐specific genomic foundation model pretrained on 149 rice genomes, illustrates that pangenomic crop data can be used to train species‐specific DNA language models that may reach performance comparable to leading multi‐species models (Yang et al., 2026). Although direct applications of artificial intelligence or machine learning in pantranscriptomics remain limited, these are expected to become increasingly relevant for interpreting large pantranscriptome resources. This includes isoform‐level annotation, genotype‐specific expression analysis (Kanwal et al., 2025), regulatory network inference and candidate gene prioritisation. This view is supported by broader developments in plant transcriptomics, where machine learning is already used for differential expression analysis, functional annotation, regulatory network reconstruction and candidate gene prioritisation (Panahi et al., 2025). If models like OryzaG3 were to be trained with data on presence or absence of specialised metabolites, correlations of genetic or transcriptomic features and metabolomics data could be made more accessible also for a wider research community. Together, these observations highlight that both methodological advances within pangenomics and pantranscriptomics and the integration of established strategies can help to unlock the full potential of these approaches for specialised metabolism research.

## CONFLICT OF INTEREST

The authors declare no conflict of interests.

## Data Availability

Data sharing is not applicable to this article as no new data were created or analysed in this study.
